# 3D Printed Models in Cardiovascular Disease: An Exciting Future to Deliver Personalized Medicine

**DOI:** 10.3390/mi13101575

**Published:** 2022-09-22

**Authors:** Zhonghua Sun, Cleo Wee

**Affiliations:** 1 Discipline of Medical Radiation Science, Curtin Medical School, Curtin University, Perth 6845, Australia; 2 Curtin Medical School, Faculty of Health Sciences, Curtin University, Perth 6845, Australia

**Keywords:** 3D printing, visualization, heart, vascular disease, model, medicine, anatomy, pathology

## Abstract

3D printing has shown great promise in medical applications with increased reports in the literature. Patient-specific 3D printed heart and vascular models replicate normal anatomy and pathology with high accuracy and demonstrate superior advantages over the standard image visualizations for improving understanding of complex cardiovascular structures, providing guidance for surgical planning and simulation of interventional procedures, as well as enhancing doctor-to-patient communication. 3D printed models can also be used to optimize CT scanning protocols for radiation dose reduction. This review article provides an overview of the current status of using 3D printing technology in cardiovascular disease. Limitations and barriers to applying 3D printing in clinical practice are emphasized while future directions are highlighted.

## 1. Introduction

**Figure 1:**
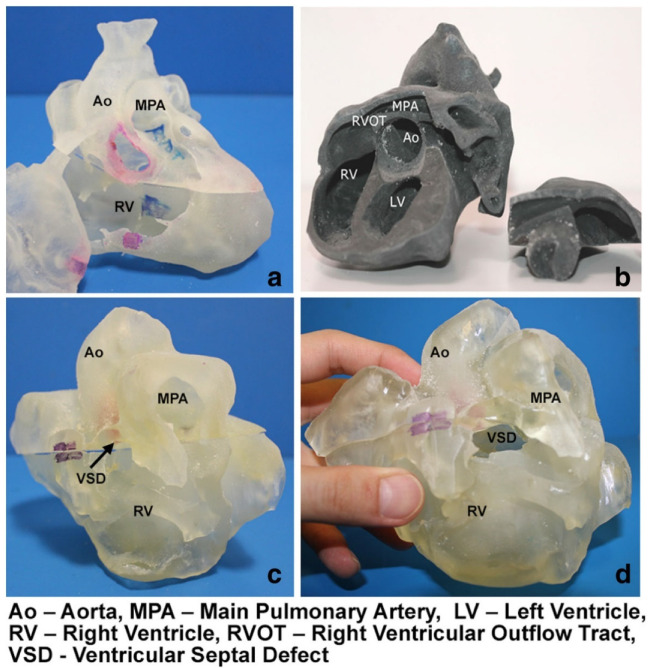
3D printed heart models showing normal anatomy and pathology. (**a**) Normal heart model created from cardiac CT and is partitioned into three pieces allowing visualization of interventricular septum. (**b**) Repaired tetralogy of Fallot (ToF) from an adult patient. The model was created from cardiac magnetic resonance imaging (MRI) and separated into two pieces allowing for clear visualization of overriding aorta and pulmonary infundibular stenosis. (**c**) Unrepaired ToF heart model from an infant. The model was created from 3D echocardiographic images and partitioned into two pieces showing the ventricular septal defect (VSD). (**d**) Unrepaired ToF heart model from an infant with superior and inferior portions showing VSD and the aortic overriding in relation to the VSD. Reprinted with permission under the open access from Loke et al. [20].

Three-dimensional (3D) printing has become an increasingly used tool in medicine, with the literature documenting its applications from the education of medical students and healthcare professionals to assisting clinical decision-making such as pre-surgical planning and simulation or intraoperative guidance and enhancing doctor-to-patient communication [1,2,3,4,5,6,7,8,9,10,11,12,13,14,15]. Patient-specific 3D printed models based on imaging datasets, most commonly using computed tomography (CT) and magnetic resonance imaging (MRI), have been proven to replicate anatomy and pathology with high accuracy when compared to the original source data (Figure 1) [16,17,18,19,20]. With the generation of high-quality and highly accurate 3D printed models, the applications have been extended to various clinical domains, with 3D printed models serving as a valuable additional tool to enhance diagnosis, surgical planning, and treatment strategies, with eventual improvements in patient outcomes [15,16,17,18,19,20].

The use of 3D printed models in maxillofacial and orthopedics is well-explored in the literature, and its value in cardiovascular disease is showing great promise and potential to change the current practice [1,7,8,11,12,13,14,16,17,18,19,20]. Increasing evidence has shown that the use of 3D printing in cardiovascular disease has overcome limitations that are inherent in the current image visualizations, thus playing an important role in many aspects, from education to clinical practice [6,7,11,16,17,18,19,20]. Patient-specific 3D printed models can enhance medical and patient education and understanding of complex anatomy and pathology by demonstrating realistic spatial relationships in various cardiovascular diseases, planning or simulating complex cardiovascular procedures, and training or guiding junior or inexperienced doctors to perform cardiovascular interventional procedures so that their confidence or skills will be improved. Further, 3D-printed personalized models can be used to optimize cardiac CT imaging protocols with the aim of reducing radiation exposure to patients. With advancements in 3D printing technology, including both printers and printing materials, it is feasible to print 3D models with materials like tissue properties of the cardiovascular system, therefore, further augmenting its applications in the cardiovascular domain. This review aimed to provide an update on the applications of 3D printed models in cardiovascular disease, with the limitations and barriers of applying 3D printed models in clinical practice identified. Future directions are also highlighted, with some potential areas for development emphasized.

## 2. Processes from Image Post-Processing to 3D Printed Models

It has been well described in the literature regarding the steps or processes to generate 3D printed models using CT or MRI (sometimes using echocardiography) imaging datasets [1,7,14]. Briefly, good quality imaging data with a high spatial resolution is an essential component for image post-processing and segmentation to create 3D volumetric data, as the quality of source data has a direct impact on the quality and accuracy of 3D printed models. This is especially important for producing 3D-printed heart or vascular models for demonstrating fine details or structures such as cardiac defects or coronary artery abnormalities [7,14].

**Figure 2:**
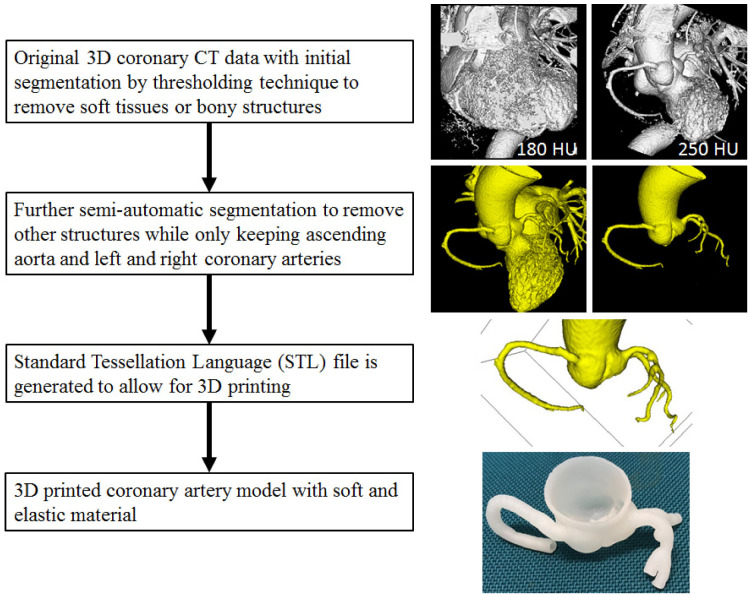
Steps to generate 3D printed models through image post-processing and segmentation of coronary CT images to extract the ascending aorta and coronary tree from the original volume data to STL file for printing 3D model. Reprinted with permission under open access from Sun [21].

Figure 2 is a flow chart showing the steps to create a 3D reconstruction model for printing a physically realistic model of a coronary artery [21]. There is no standard requirement for software tools to perform images segmentation, although commercially available software such as Mimics (Materialise Leuven, Belgium), MeVislab (Mevismedical Solutions, Bremen, Germany) and Analyze 12.0/14.0 (AnalyzeDirect, Inc, Lexana, KS, USA) are the commonly used packages for image post-processing and segmentation. Open source software tools such as 3D Slicer and ITK-SNAP are reported to create a 3D printed heart and vascular models with high accuracy [22].

## 3. 3D Printers and Printing Materials

Once image segmentation is done, the Standard Tessellation Language (STL) file is sent to a 3D printer for printing a physical model. Several 3D printers and printing materials are available in the market, allowing for printing either flexible or rigid cardiovascular models, depending on the preference of the nature of the study. Fused deposition modeling (FDM), Stereolithography (SLA), selective laser sintering (SLS) and Polyjet printers are commonly used in most of the studies and readers are referred to several nice review articles for details [1,7,21,23].

**Figure 3:**
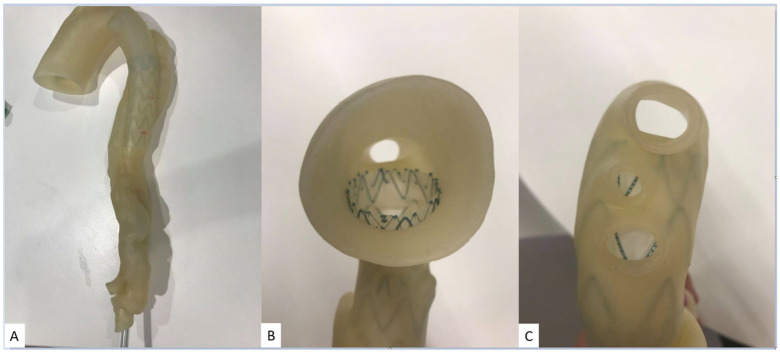
Stent graft deployed in 3D printed model. The aortic model was printed with Visijet CE-NT A30. (**A**): Deployed stent graft visible through model wall. (**B**): Axial view from proximal aortic arch. (**C**): Caudal view down aortic arch vessels. Reprinted with permission under the open access from Wu et al. [25].

The selection of appropriate printing materials is also important to ensure that the 3D printed heart and vascular models serve the purpose of different utilizations. In addition to printing multi-color models, mechanical properties need to be considered for producing realistic cardiovascular models to simulate similar tissue properties in terms of modulus of elasticity. In previous studies, TangoPlus FLX930, TangoGray ^TM^ and TangoBlack ^TM^ (Stratasys) are reported to produce 3D printed heart and vascular models with soft and elastic features [24]. Recent research has shown that Visijet CE-NT (3D Systems Inc., Wilsonville, OR, USA) has the same tissue properties as cardiac tissues with similar CT attenuation; thus, it is more suitable for the 3D printed heart and vascular models (Figure 3) [25].

## 4. Education Value of 3D Printed Models in Cardiovascular Disease

3D printed models are increasingly used in medical education, with promising results achieved when compared to traditional teaching methods. Studies have shown its educational value in two areas as assessed by medical students and clinicians (cardiothoracic surgeons, cardiologists, cardiac imaging specialists including radiologists and radiographers, residents or registrars, and clinical nurses) [26,27,28,29,30,31,32,33,34,35,36,37,38,39]. Table 1 summarizes studies reporting the medical education of 3D printed models compared to traditional teaching methods or other advanced tools.

### 4.1. Medical Student Education

**Figure 4:**
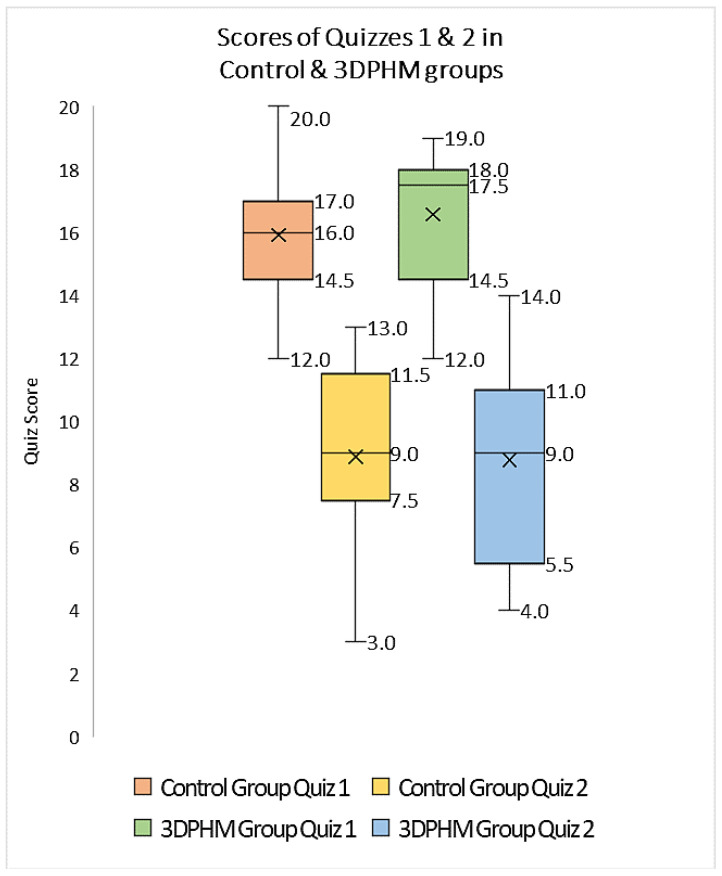
Boxplot of the scores (out of 20) achieved by 3D printing and control groups in Quiz 1 and Quiz 2. 3DPHM-3D printed heart model. Reprinted with permission under open access from Lau and Sun [27].

Several studies based on randomized controlled trials (RCTs) and cross-sectional or cohort studies have documented the educational value of 3D printed models in cardiovascular anatomy and pathology [20,26,27,28,29,30,31]. Most of these studies proved the significant improvements in students’ knowledge and understanding of both normal cardiac anatomy and pathology (mainly in congenital heart disease) with the use of 3D printed models over the current teaching methods such as using 2D or 3D diagrams, cadavers and educational lectures. Three-dimensional printed heart and vascular models were shown to increase students’ confidence in recognizing cardiac anatomical structures and congenital anomalies. One recent study investigated whether 3D printed heart models improved the immediate and long-term knowledge retention among medical students when compared to the current teaching methods [27]. Authors delivered an education workshop comprising 2D cardiac CT images and 3D digital models to both control and 3D printing groups (53 second- and third-year medical students), while the 3D printing group received 3D printed models as additional components. Four types of congenital heart disease (CHD) were presented to the medical students who completed an online quiz at the end of the session and another online quiz 6 weeks later. The results showed no significant improvements in both immediate knowledge and knowledge retention with the use of 3D printed CHD models, despite slightly higher scores obtained in the 3D printing group than in the control group (Figure 4).

### 4.2. Clinician Education

Due to the complexity and wide heterogeneity of CHD lesions, it is difficult to fully understand the complex 3D anatomy and pathology on a 2D flat screen, thus rendering 3D printed models a valuable tool for the education of clinicians or healthcare professionals. Most of the studies reported the educational value of using 3D printed CHD models in pediatric or medical residents [32,33,34,35,36], with some involving pediatric cardiologists [35] (Table 1). There is consistent agreement among these studies that 3D printed models significantly increased the participants’ knowledge of cardiac anatomy and CHD pathology when compared to conventional education or imaging approaches. Significantly higher scores were achieved in the 3D printing groups than those in the control groups, and this is especially apparent when complex CHD was involved [36,39], and 3D printed models were rated as an excellent tool for anatomy teaching, as well as improving diagnostic rate assessed by both experts and students [39].

Interestingly, a recent study by Lau et al. compared 3D printing with virtual reality (VR) technologies in 4 selected CHD cases among 29 participants [32]. All participants received a 15-min session of 2D/3D visualizations plus 3D printed CHD models and VR. There was no significant difference between these two tools in medical education and preoperative planning of CHD. Thus, this study highlights the potential value of using VR in combination with 3D printing technology in medical education. In addition to VR, advanced innovative tools, including augmented reality (AR) and mixed reality (MR), have also been applied in medical education by providing immersive learning experiences that may enhance the teaching and learning of complex content such as cardiovascular anatomy and pathology [40,41,42,43]. Barteit et al. conducted a systematic review of VR, AR and MR in medical education through an analysis of 27 studies comprising 956 participants [44]. Medical students (59.9%) and residents represented (30.2%) most of the participants with AR and MR mainly implemented in surgery training (48%) and anatomy learning (15%). This review and other research studies highlighted the effectiveness of using VR, AR or MR tools in teaching cardiac anatomy and pathology [42,43,44]. The use of 3D-printed physical models, along with these novel visualization tools, will further enhance medical education.

Two studies investigated the educational value of 3D printed models for cardiac nurses or nursing students. Tan et al., in their RCT, randomly allocated 132 nursing students of congenital heart surgery to the 3D printing or traditional groups to assess their knowledge and critical thinking of a real case of atrial septal defect [37]. Significant higher scores were found in the 3D printing group than those in the traditional group regarding students’ comprehensive thinking ability of cardiovascular anatomy and congenital heart disease (Table 2). Biglino et al. showed that the 3D printed heart and CHD models enhanced cardiac nurses’ knowledge of understanding cardiac anatomy and pathology [38]. There was no significant difference regarding the usefulness of 3D printed models in understanding different cardiac defects.

## 5. Clinical Applications of 3D Printed Models in Cardiovascular Disease

Clinical applications of using 3D printed models in cardiovascular disease are manifested in four areas: assisting pre-surgical planning of complex cardiac surgery procedures, simulation of surgical or interventional radiology procedures for medical residents or trainees, improving doctor-to-patient communication and development of optimal CT scanning protocols for reduction of radiation dose. The following sections will review these applications based on the current research studies.

### 5.1. Pre-Surgical Planning of Complex Cardiac or Cardiovascular Procedures

Application of 3D printed models in pre-surgical planning of complex cardiac or cardiovascular procedures represents the most common applications, and this is shown in a recent review article with nearly 50% of the applications on the assessment of the clinical value of 3D printed models in pre-surgical planning or simulation of cardiovascular procedures [45,46]. Table 3 summarizes some recent studies based on single and multi-center reports on the clinical value of 3D printed models in pre-surgical planning, with most of them focusing on CHD surgeries [24,47,48,49,50,51,52]. The multi-center study by Valverde et al. is a well-recognized report involving ten international centers with the inclusion of 40 complex CHD cases. In nearly half of the cases (47.5%), a surgical decision was changed with the use of 3D printed heart models, while in 28 cases conventionally considered for surgery, the surgical approach was modified in 15 cases (53.6%) after evaluating the 3D printed models [47].

Three single-center studies documented their three years and eight years of experience in using 3D printed models in CHD surgeries with the creation of more than 100 models [22,50,52]. Gomez-Ciriza and colleagues developed 138 affordable 3D printed models (an average cost per model is EUR85.7) and presented similar findings as Valverde et al., with 47.5% of surgical planning modified with the use of 3D printed models when compared to the original surgical plan. Further, these 3D printed models were scored useful for communicating with patients and parents when assessed by cardiac surgeons and pediatric cardiologists [22]. Ryan et al. reported their three years of experience with the generation of 164 models for a range of purposes [50]. When compared to the standard of care pre-procedural planning, 3D printed models reduced the operating length of time, 30-day mortality and readmission rate, although this did not reach statistical significance. Ghosh et al. reviewed the growth and development of using more than 100 3D printed models in their practice over a period of 3 years, with 96 of the models used for operative planning of CHD cases. Their experience shows that 3D printing can be incorporated into the pre-procedural planning of CHD in a pediatric clinical center [52].

Other studies based on case series or case reports (<30 cases) from a single-center experience showed consistent findings that 3D printed models assisted pre-surgical planning and simulation of CHD and cardiomyopathy (Table 3). Russo et al. applied 3D printed models of aortic stenosis to simulate transcatheter aortic valve replacement (TAVR) for predicting the risk of developing coronary artery obstruction or complications. The simulation results of 3D printed models correlated well with clinical outcomes and thus can be used to plan TAVR procedures for reducing potential risks or complications [53].

Lau et al. further compared 3D printed models with VR in the clinical value of these two tools in preoperative planning and education of CHD [32]. Interestingly, both VR and 3D printed models are useful for understanding complex CHD conditions and preoperative planning when compared with the standard 2D or 3D visualizations, although VR scored higher than 3D printed models with no significant difference. Similar findings were reported by Chen et al. with VR and MR using Hololens enhanced understanding of intracardiac anatomy when compared to 3D printed models [48]. This could highlight the potential value of using VR/MR in pre-surgical planning of cardiovascular disease when a 3D printing facility is not available or using a combination of both methods [44].

### 5.2. Simulation of Surgical or Interventional Procedures

The use of 3D printed models in simulating cardiac surgeries or interventional procedures is another area that has been well explored in the literature, with promising results reported. Table 4 summarizes some representative studies documenting the value of using 3D-printed personalized models in this field.

One of the common applications of 3D printing lies in guiding left atrial appendage occluder device selection to improve treatment outcomes and reduce potential complications associated with LAA occluder procedures. RCT, case-control and cross-sectional studies have shown the significant advantages of 3D printing guided procedures over traditional methods based on imaging (CT, echocardiography or intraoperative angiography), with key findings, including: good agreement between 3D printed model-based sizes and finally implanted occluder device sizes; reduced procedure time with no major adverse events or mortality and reduce radiation exposure to patients when compared to the control group without having 3D printed models [54,55,56,57] (Table 4). Further, it seems the clinical value of 3D printed models depends on the type of occluder devices, as the correlation between 3D printed models and inserted device size was different for different occluders [58]. This may need to draw attention to clinicians when choosing different devices in planning the treatment of LAA.

**Figure 5:**
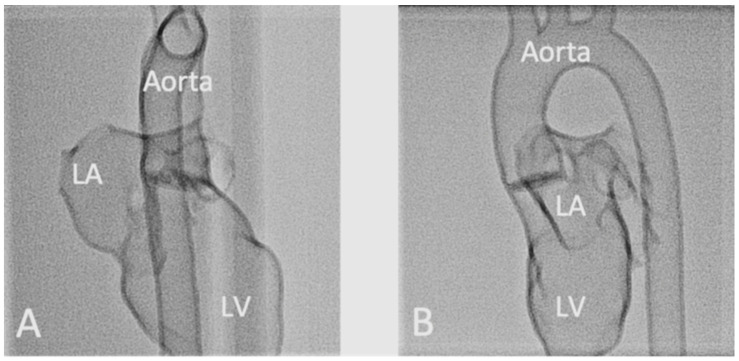
Influence of the projection levels on the anatomic representation during fluoroscopy of a 3D printed left heart model. Representation of a 3D printed heart model in anteroposterior (**A**) and lateral views (**B**). LA-left atrium, LV-left ventricle. Reprinted with permission under the open access from Brunner et al. [65].

**Figure 6:**
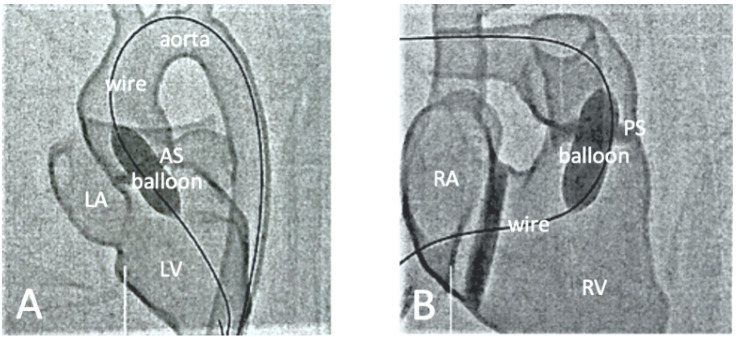
Fluoroscopic documentation of balloon dilatation of valvular stenosis with the 3D printed heart model. (**A**): Balloon dilatation of a valvular aortic stenosis. The inflated balloon is positioned at the level of the aortic valve. The long guidewire is inserted via the descending aorta with its tip lying in the LV. (**B**): Balloon dilatation of a valvular pulmonary stenosis. The inflated balloon is positioned at the level of the pulmonary valve. The long guide wire is inserted via the inferior vena cava through the RA into the RV, with its tip lying in the right pulmonary artery. AS-aortic stenosis, PS-pulmonary stenosis, LA-left atrium, LV-left ventricle, RA-right atrium, RV-right ventricle. Reprinted with permission under the open access from Brunner et al. [65].

Another common application of 3D printing is to simulate cardiac or cardiovascular procedures, in particular, interventional cardiology or radiology procedures, for training surgeons or trainees to perform complex or challenging procedures. The use of personalized 3D printed vascular models aims to increase the confidence and surgical skills of surgeons prior to operating on real patients [59,60,61,62,63,64,65]. Endovascular aneurysm repair (EVAR) is a widely used, less invasive procedure for treating aortic dissection and aneurysm, and the use of 3D printed models in simulating EVAR procedures has significantly reduced fluoroscopy time, procedure time, contrast medium and cannulation time when compared to the control group using standard training approach [60,63,66,67,68,69]. Similar findings are also reported by a recent study on the use of 3D printed CHD models for a hands-on training program to simulate interventional cardiology procedures [65] (Figure 5 and Figure 6).

Printing materials have an impact on the user’s performance when performing a simulation of interventional procedures as 3D models printed with soft and elastic materials allow the user to acquire a similar tactile experience to human vascular tissue, and this is especially important for structures like vessels and cardiac valves as highlighted in some studies [64,65]. Current developments in 3D printing technologies and printing materials have enabled achieving this goal with the selection of appropriate materials to replicate human cardiovascular tissue properties [25,70]. Evidence strongly recommends that 3D printed vascular models serve as a valuable tool for simulating and rehearsing cardiac or interventional radiology procedures.

**Figure 7:**
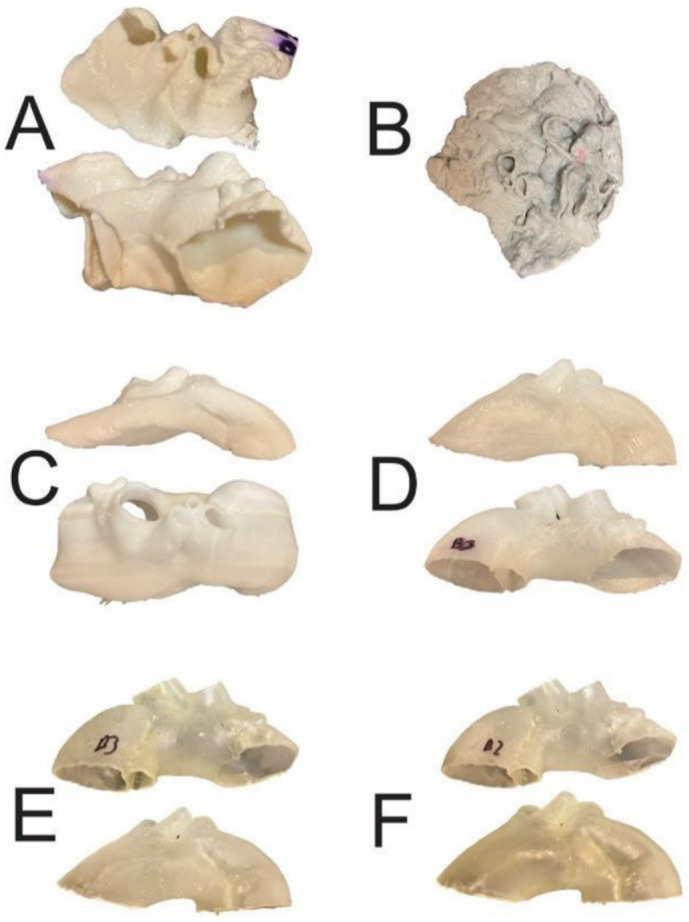
The 3D aortic templates after sterilization in 121 °C autoclave. Templates made of PLA (**A**), PETG (**B**), and PP (**C**) were affected by significant deformations, whereas those made of nylon (D), rigid (**E**) and flexible resins (**F**) were intact. PLA-polylactic acid; PETG-polyethylene terephthalate glycol; PP-polypropylene. Reprinted with permission under the open access from Rynio et al. [66].

Rynio et al., in their recent study, tested the effect of sterilization on 3D printed aortic templates, which are used in aortic stent grafting. They chose a complex case of aortic arch dissection and printed 11 models with use of six common materials. The sterilization was performed under three different methods and temperatures to determine the change of 3D printed model geometry and dimensions. Figure 7 shows the effect of high temperature on the deformation of 3D printed aortic templates printed with different materials [66]. This study presented important findings on the relationship between 3D printing materials and sterilization methods.

### 5.3. Enhancement of Doctor-to-Patient Communication

**Figure 8:**
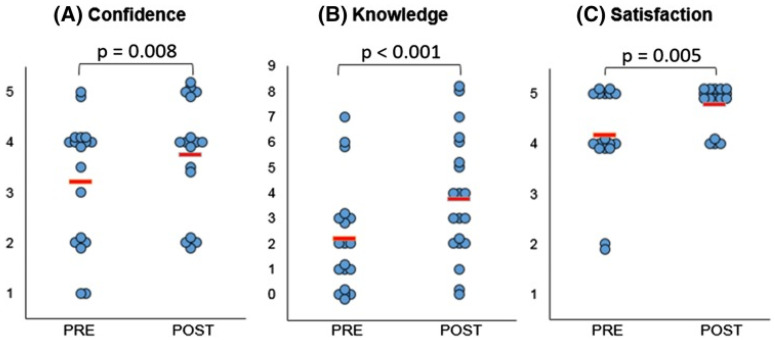
Statistically significant differences were noted in confidence (**A**), knowledge (**B**) and satisfaction (**C**) amongst participants, comparing responses before (“Pre”) and after (“Post”) their medical consultation. (**A**): One refers to not at all confident, and five to very confident. (**B**): Each point represents a point in knowledge, as marked based on the correct name of primary diagnosis, correctly identified keywords and correct use of diagrams. (**C**): One indicates very dissatisfied, and five is very satisfied. The red lines indicate average score. Reprinted with permission under the open access from Biglino et al. [72].

**Figure 9:**
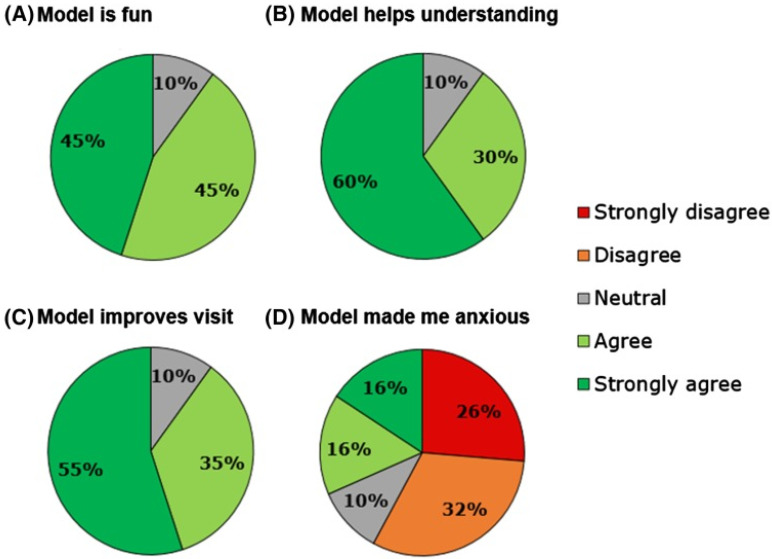
Participants’ response to different statements on the usefulness of 3D printed models. Reprinted with permission under the open access from Biglino et al. [72].

The role of using 3D printed models in enhancing doctor-patient communication has been reported in the literature, although only a limited number of studies are available, according to a recent scoping review. Traynor et al. conducted a comprehensive review of the current literature and identified 19 studies on the use of 3D printing in patient communication [71]. Of these studies, seven studies were related to cardiology and cardiovascular surgery [49,72,73,74,75,76,77], of which four studies were reported from the same research group [72,73,74,75]. Biglino and colleagues investigated the impact of 3D printed CHD models on communication from the perspectives of different stakeholders, including clinicians (cardiologists), patients and the parents of patients. Results of these studies presented consistent findings that 3D printed models facilitated communication with colleagues, patients and parents, with a significant improvement in patients′ and parents’ knowledge or understanding of the disease condition, or satisfaction [74] (Figure 8 and Figure 9). The other three studies also supported the improvement between doctor-patient or between clinicians’ communication with the use of 3D printed models heart models [49,76,77]. More research needs to be done on patient engagement in decision-making through understanding their disease conditions by use of 3D printing.

### 5.4. Development of Optimal CT Scanning Protocols

Application of 3D printed models in developing optimal CT scanning protocols is a new research direction with only a few studies available in the literature, but with promising results achieved. Cardiac CT is a widely used modality for diagnostic assessment of cardiovascular disease, most used in coronary artery disease, aortic aneurysm or dissection and pulmonary embolism [78,79,80]. The main concern of cardiac CT is a high radiation dose; hence, the use of appropriate CT scanning protocols is clinically important for the reduction of patient radiation exposure without compromising image quality. Despite commercially available anthropomorphic human phantoms for dose reduction and image quality experiments, they are not only expensive but do not represent an individual patient’s situation due to the use of average adult or pediatric body sizes. The 3D-printed personalized models based on CT images overcome these limitations by producing individualized heart or cardiovascular models for studying CT protocols.

**Figure 10:**
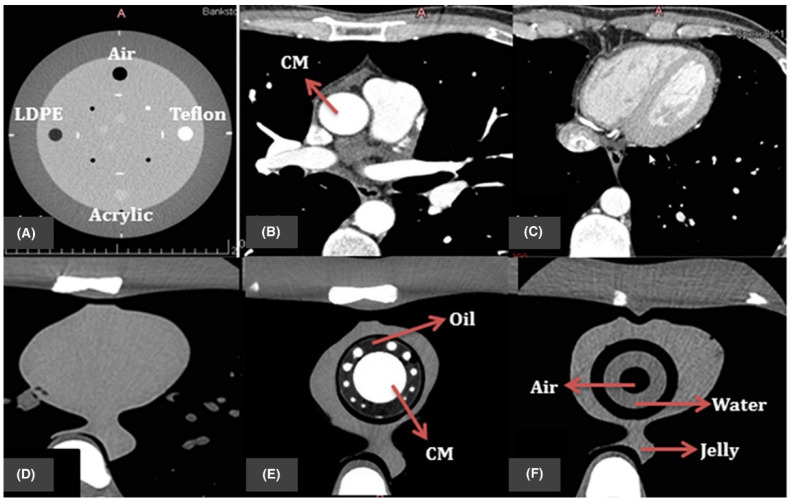
The resulting axial CT of (**A**) four inserts in Catphan@ 500 phantom; (**B**,**C**) patient image datasets for cardiac CT; (**D**) original cardiac insert of anthropomorphic chest phantom; (**E**,**F**) 3D printed cardiac insert phantom with contrast materials (CM), oil, air, water and jelly segmented labeled. Reprinted with permission under the open access from Abdullah et al. [81].

**Figure 11:**
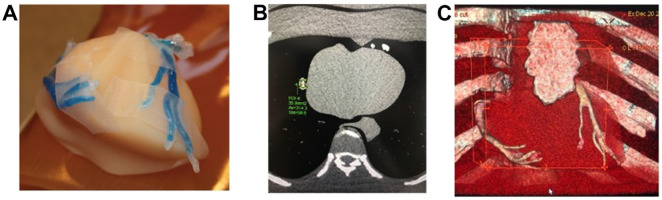
(**A**): Contrast-filled 3D printed coronary arteries on the Lungman. (**B**): CT image of the coronary arteries. (**C**): 3D volume rendering. Reprinted with permission under the open access from Morup et al. [82].

Several studies have explored the feasibility of developing 3D printed heart and vascular models for CT dose optimization [81,82,83,84,85,86,87,88,89,90]. Abdullah et al. developed an organ-specific cardiac insert phantom for the investigation of cardiac CT scanning protocols. Although it does represent the novelty of the study design with the simulation of surrounding cardiac structures as well as contrast medium during cardiac CT scan (Figure 10), the study lacks personalized 3D printed anatomical structures [81]. Morup et al. tested four different materials (gelatin mixtures, pig hearts and Ecoflex^TM^ silicone) with the aim of determining the appropriate one to simulate human heart and vascular tissue properties. Their results showed that the contrast-filled Ecoflex ^TM^ silicone had a mean CT attenuation of 318 Hounsfield Units (HUs) which is close to the contrast enhancement CT attenuation in real patients (Figure 11), serving as a cost-effective model for CT protocol optimization [82].

**Figure 12:**
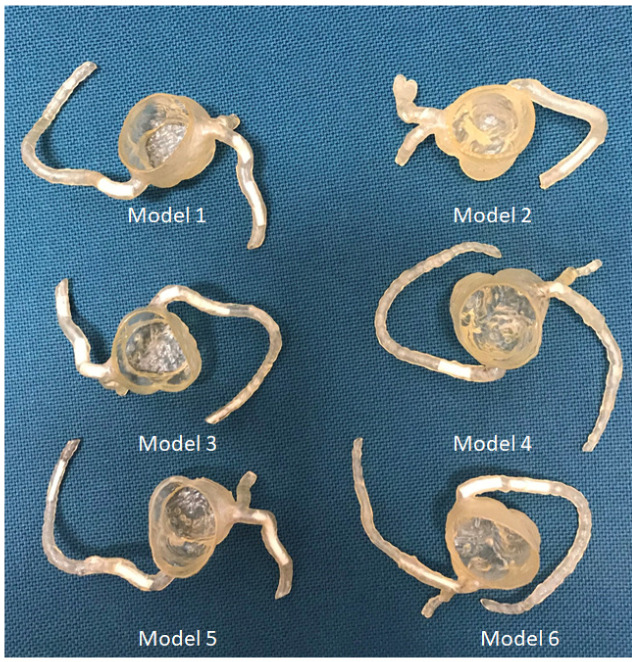
The 3D-printed coronary artery models with simulated calcified plaques were inserted into the coronary artery branches. Reprinted with permission under the open access from Sun et al. [83].

**Figure 13:**
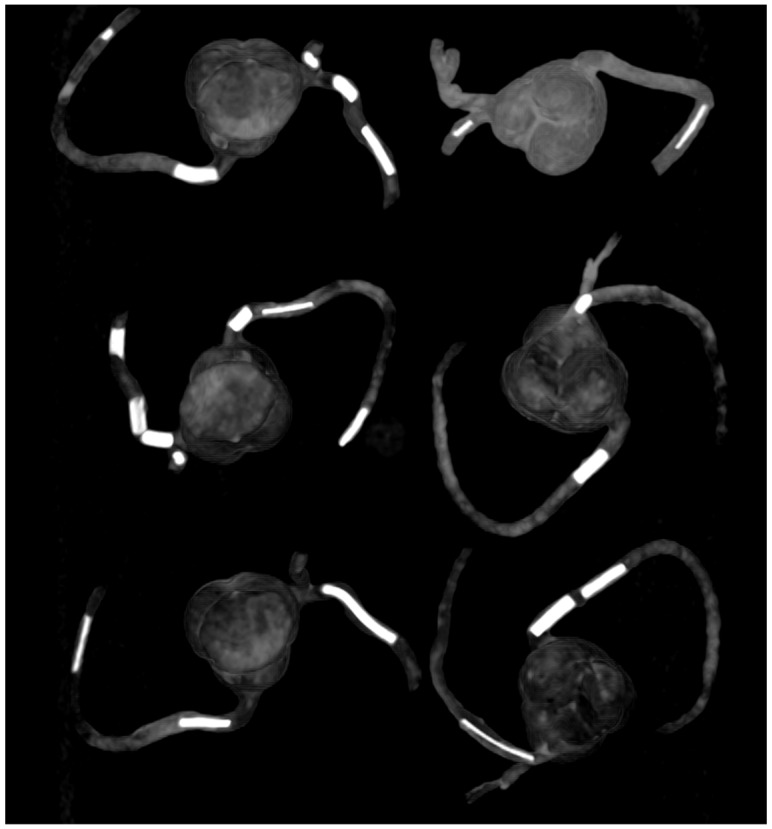
Coronal maximum-intensity projection images showing the calcified plaques (with different diameters and lengths) in six 3D printed coronary models. Reprinted with permission under the open access from Sun et al. [83].

We have developed personalized coronary artery models and aorta models using CT images for optimizing CT protocols and visualization of coronary calcified plaques and stenting [83,84,85,86]. Calcified plaques were created using a mixture of silicone, ethiodized oil and carbonate to simulate calcification in the coronary arteries. The combination of silicone and 32.8% calcium carbonate was found to produce CT attenuation of 800 HU, representing extensive calcification (Figure 12); thus, it is suitable for studying CT protocols for assessing calcified coronary plaques (Figure 13) [83].

**Figure 14:**
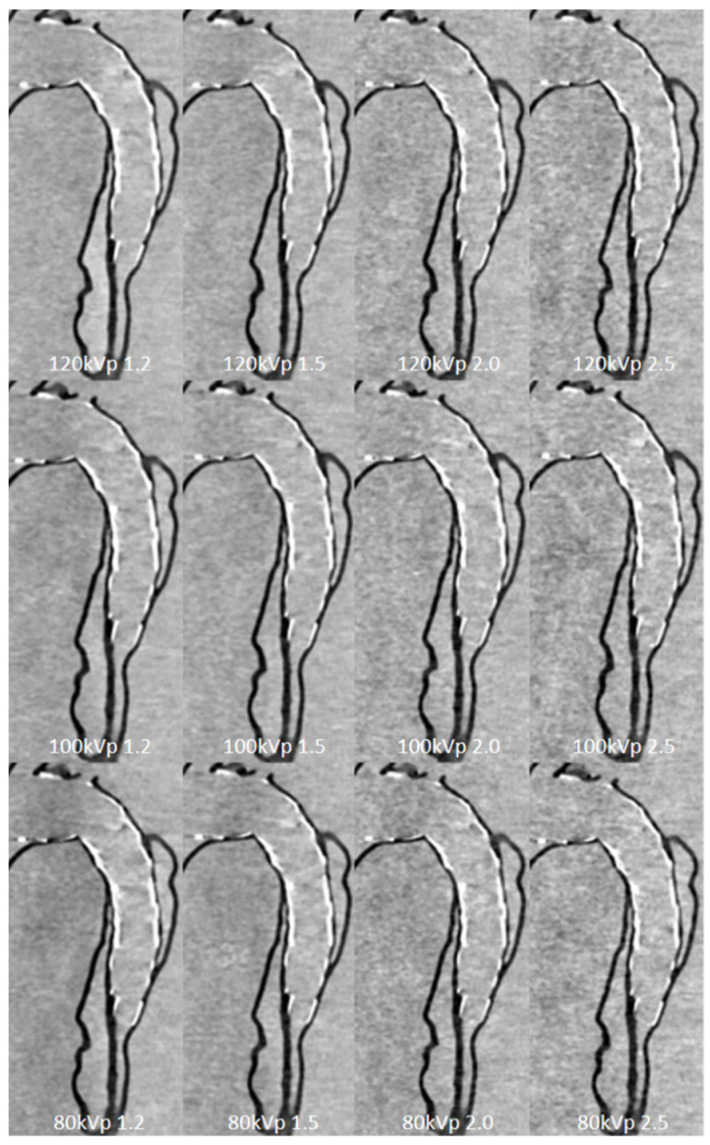
Sagittal reformatted images of the CTA protocols. When kVp decreased to 80, image noise increased with the use of high-pitch value protocols such as 2.0 and 2.5. Reprinted with permission under the open access from Wu et al. [25].

Further, 3D printed models of type B aortic dissection were developed using CT datasets to replicate true lumen, false lumen, aortic aneurysm, and insertion of the aortic stent graft to simulate EVAR (Figure 3) [25]. CT scans were conducted to investigate the optimal aortic CT angiography (CTA) protocols in the follow-up of patients with aortic dissection following EVAR treatment. Low kilovoltage peak (80 kVp) and high pitch (2.0) can be suggested as the optimal CT protocol with a reduction of more than 20% radiation dose without affecting image quality as assessed quantitatively and qualitatively (Figure 14) [25,88]. Since aorta CTA is the preferred imaging modality for both diagnosis of aortic aneurysm/dissection and follow-up of EVAR patients due to consecutive CT scans at regular periods, low-dose CT protocol is of paramount importance to minimize radiation dose. Thus, the use of 3D printed personalized aorta models will have great potential in the future to optimize current CT scanning protocols.

Another area of using a personalized 3D printed model lies in the simulation of pulmonary embolism with the model scanned using a range of CT pulmonary angiography protocols. Significant dose reduction by up to 80% was achieved with the use of low dose (70–80 kVp) and high pitch (3.2) with acceptable image quality when assessing pulmonary embolism, either in the main or side branches [89,90]. The main limitation of these studies is the lack of placing the 3D printed model in a realistic chest phantom with surrounding lungs, bones or cardiac structures. This needs to be addressed in future experiments.

## 6. Limitations, Barriers and Future Directions

Over the last decades, there have been significant advancements in the use of 3D printing technology in cardiovascular disease, with reports showing great potential in clinical applications, as well as medical education and other areas. However, there exist some limitations and barriers that need to be considered when promoting 3D printing applications in cardiovascular disease. First, despite increasing reports in the literature, robust studies are still lacking, with the majority of the current studies based on case series or relatively small sample sizes (Table 1, Table 3 and Table 4). Further, follow-up studies of the mid to long-term outcomes of how 3D printed models contribute to education and clinical practice are scarce. Second, technological improvements have enabled the printing of realistic heart and vascular models with high accuracy in replicating anatomy and pathology (including complex conditions such as CHD); however, most of the current 3D printed models are static ones, and they do not really represent real cardiovascular circulatory physiology. Future research will need to address this limitation by developing more realistic 3D printed models, with 3D printed models connected to a fluid pump with the simulation of cardiac pulse sequences, as shown in Karkkainen and other studies [60,87]. Third, the use of appropriate printing materials plays an important role, especially in the simulation of cardiac or interventional procedures, such as the operators need to experience a similar feeling when performing the simulation on 3D printed models so that clinicians and students can gain confidence and skills that are required to operate on real patients. This is already achievable with the current printing materials [25,63,70,88].

The main barriers to implementing 3D printing technology in routine cardiovascular practice are the relatively high costs associated with 3D printing (including image post-processing and segmentation) and the slow turnaround time. The first barrier will be addressed by using artificial intelligence, such as machine learning (ML) or deep learning (DL), to enhance the image segmentation process [91,92,93]. With printers available at clinical sites, the use of 3D printing technology in daily practice will become possible, and clinicians can incorporate 3D printed models into their diagnosis and decision-making process.

**Figure 15:**
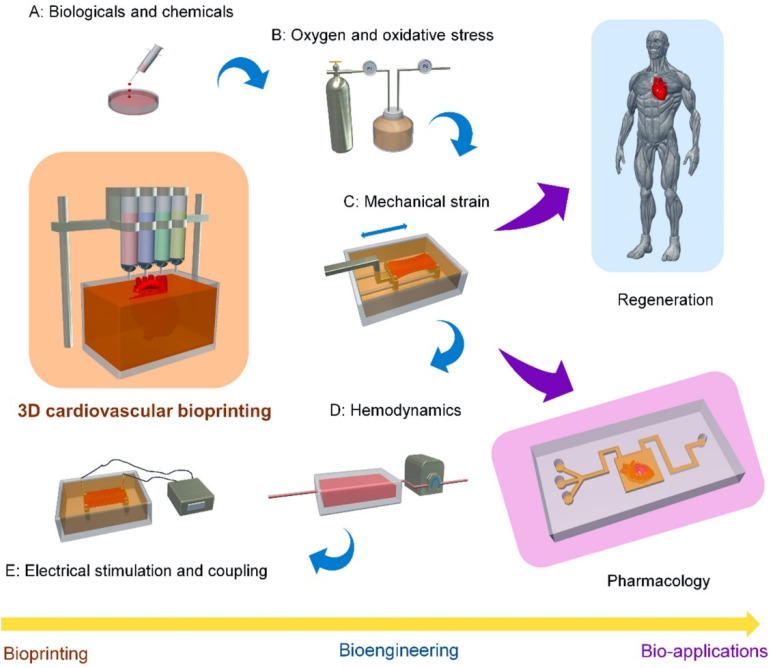
Schematic diagram outlining the techniques for 3D cardiovascular bioprinting, bioengineering methods, and bio-applications in regeneration and pharmacology. Reprinted with permission from Cui et al. [94].

Bioprinting is the future of 3D printing technology in medical applications, with significant progress made in printing cardiovascular constructs, cardiovascular regeneration and pharmacology over the last decades [94,95,96,97,98,99]. Figure 15 outlines the scheme of 3D bioprinting technology in the cardiovascular system. Despite promising results available in the literature, current technologies and printing materials limit the application of bioprinting to only small units of functional cardiovascular tissues. There are still many challenges to be resolved before printing complete organs, such as a fully functional heart, becomes possible [94].

A close collaboration between clinicians and academic researchers is essential to achieve the goal that clinicians are aware of the 3D printing technologies and capabilities so that their knowledge, skills and confidence in using the 3D printed personalized models are enhanced. This will play an important role in ensuring the incorporation of 3D printing technology into routine practices to benefit diagnostic strategy and clinical decision-making [100].

## 7. Summary and Concluding Remarks

There is no doubt that 3D printing technology has revolutionized our current practice (both education and clinical) in the diagnosis and management of patients with cardiovascular disease. The 3D printed models assist surgical planning and the simulation of cardiac procedures, thus greatly improving understanding of anatomy and pathology, increasing clinicians’, especially trainees’, knowledge, skills and confidence when performing operations on patients. This has a significant clinical impact on training young and junior doctors with 3D printed personalized models. Simulation of cardiac or interventional procedures will lead to high operation success rates with few complications, thus improving patient outcomes. 3D printed models serve as a valuable education tool for medical students or graduates in learning cardiovascular anatomy and pathology; therefore, they could be used as an alternative to the current teaching methods.

An emerging area of using personalized 3D printed models is to optimize CT scanning protocols with evidence showing feasibility and promise. More research could be developed along this pathway to optimize current CT practice with the delivery of low-dose protocols. With the further development of 3D printing technology and the incorporation of other technologies, such as ML and DL and VR, AR and MR, a combination of using advanced technologies will be of great benefit to our healthcare through the delivery of personalized medicine.

## Figures and Tables

**Table 1:** Usefulness of 3D printed models in medical and clinical education.

Author	Study Design	Sample Size and Participants	Original Data Source	Application in CVD	Image Processing Software	3D Printer/ Printing Parameters	3D Printing Material	Key Findings
Garas et al. [26]	Cross-sectional study	Twenty-three students from first-year health science courses (*n* = 11) and third-year human biology preclinical courses (*n* = 12). All participants were exposed to plastinated, 3D-printed models and cadaverous specimens.	MRI and CT	3D printed models of heart, shoulder and thigh	NR	NR	Resin	3D printed models have the potential to assist students in anatomy learning when compared to traditional teaching methods: 3D printed models were scored to be easiest to identify anatomical features than others (*p*<0.001). 3D printed models received the highest percentage of the preferred method of learning anatomy compared to cadavers and plastinated models (45% vs. 36% and 18%, *p* < 0.001).
Lau & Sun [27]	Cohort study	Fifty-three second-year (*n* = 48) and third-year (*n* = 5) medical students, with 28 in the 3D printing group and 25 in the control group. The control group received an education workshop, digital 3D models and 2D images, while the 3D printing group received all the above plus 3D printed models.	CT	Four types of CHD: ASD, VSD, ToF and DORV	Mimics Innovation Suite 22.0 (Materialise, Leuven, Belgium)	Formlabs (Somerville, MA, USA) & Fabbxible Technology (Pulau Pinang, Malaysia). A 1 mm-thick shell was added to the digital model.	Flexible V4 Resin & TPU80A	3D printed models do not significantly improve both immediate knowledge acquisition and knowledge retention among the medical students, although 3D printed model group had slightly higher scores than the control group (7.79 ± 2.63 vs. 7.04 ± 2.64). 3D printed models were ranked to increase their knowledge in CHD, but no significant difference compared to 2D/3D views (*p* > 0.05).
Karsenty et al. [28]	Randomized controlled trial	Three hundred forty-seven fifth-year medical students. Students were randomized into printing groups or control groups. All students received the same 20 min lecture with projected digital 2D images. The 3D printing groups also received 3D printed models during the lecture.	CT	CHD includes ASD, VSD, CoA and ToF	Mimics and 3-Matic (Materialise, Leuven, Belgium). The mean time from segmentation to printing: ASD and CoA: 15 min VSD: 30 min ToF: 60 min	Stream 20 pro printer (Volumic, France)	Biodegradable polylactic acid (PLA) filament	Objective knowledge improvement after the lecture was higher in the 3D printing group compared to the control group (16.3 ± 2.6 vs. 14.8 ± 2.8 out of 20, *p* < 0.0001). Similar results were observed for each CHD for post-lecture scores (*p* = 0.0001 ASD group; *p* = 0.002 VSD group; *p* = 0.0005 CoA group; *p* = 0.003 ToF group). Students’ opinion of their understanding of CHDs was higher in the 3D printing group compared to the control group (respectively 4.2 ± 0.5 vs. 3.8 ± 0.4 out of 5, *p* < 0.0001).
Lim et al. [29]	A double-blinded randomized controlled trial	Fifty-two first-year medical students were randomly assigned to three groups who underwent self-directed learning sessions using either cadaveric materials (*n* = 18), 3D prints (*n* = 17), or a combination of cadaveric materials/3D prints (combined materials) (*n* = 18).	CT	3D prints of heart and great vessels: 2 × 3D printed prosected heart with great vessels 2 × 3D printed heart without great vessels 3 × 3D printed coronary artery models	NR	NR	NR	3D printed models did not disadvantage students relative to cadaveric materials. Post-test scores were significantly higher for the 3D printing group compared to the cadaveric materials or combined materials groups (mean score of 60.83 vs. 44.81 and 44.62, *p =* 0.010). A significant improvement in test scores was detected for the 3D printing group (+18.4%, *p* = 0.003) but not for the other two groups (+8.06%, *p* = 0.083, +7.45%, *p* = 0.080).
Smerling et al. [30]	Cross-sectional study	Forty-five medical students were rotated through four different teaching stations: Station 1: embryology video Station 2: 2D diagrams Station 3: Pathology specimens Station 4: 3D printed models.	CT	3D printed cardiac models were created for the following lesions: pulmonic stenosis (PS), ASD, ToF, d-TGA, CoA and HLHS	Mimics (Materialise, Leuven, Belgium), Meshlab	Uprint SE printer (Stratasys, Eden Prairie, MN, USA)	NR	The use of 3D printed models significantly improved students’ knowledge for every pathology (mean improvement ± SD): PS (0.3 ± 0.6, 3.52, *p* < 0.001), ASD (0.6 ± 0.5, 7.69, *p* < 0.001), TOF (0.8 ± 0.5, 10.08, *p* < 0.001), d-TGA (0.9 ± 0.7, 8.70, *p* < 0.001), CoA (0.8 ± 0.6, 8.93, *p* < 0.001), HLHS (1.1 ± 0.7, 11.05, *p* < 0.001). Students “strongly agreed” the 3D printed models made them more confident in explaining congenital cardiac anatomy to others (4.23 ± 0.69), and they strongly recommended the use of 3D models for future educational sessions (mean = 4.40 ±0.69).
Su et al. [31]	Randomized controlled trial	Sixty-three medical students were randomly allocated to two groups (32 students in the experimental and 31 in the control group). Each group attended a teaching seminar on VSD, with the experimental group receiving 3D printing as an additional component	CT	3D printed models of 3 subtypes of VSD (perimembranous, subarterial and muscular).	NR	SL600 3D printer (ZRapid Tech, Wujiang, China)	Plastic material	The test results were statistically significantly higher in the experimental group (*p* = 0.02–0.03). The inter-group differences in both questionnaire and test results were significantly higher in structural conceptualization (*p* = 0.03) but not in knowledge acquisition (*p* = 0.06–0.09). Open-ended responses in the questionnaire showed the advantages of using 3D models over traditional teaching methods.
Lau et al. [32]	Cross-sectional study	Twenty-nine participants: cardiac radiologist (1), interventional radiologist (1), general radiologists (3), radiology registrars (4), sonographers (3), radiographers (16) and student radiographer (1).	CT	Four 3D printed CHD models: ASD, VSD, ToF and DORV. All participants attended 15-min demonstration session of 3D printed models and VR.	Mimics 22.0 (Materialise, Leuven, Belgium)	NR. A 1 mm-thick shell was added to the digital model.	Thermoplastic polyurethane (TPU) and flexible resin.	No significant difference was found between 3D printed models ad VR in medical education and preoperative planning of CHD (*p* > 0.05). Further, subgroup analysis between doctors and non-doctors did not reach a significant difference in these two areas regarding the value of these two technologies (*p* > 0.05).
Jones & Seckeler [33]	Randomized controlled trial	Thirty-six pediatric and combined pediatric and emergency medicine residents were randomized into interventional (n = 17) and control groups (n = 19). Each group received the 20-min lecture on vascular rings and slings, while the intervention group received 3D printed models.	CT & MRI	3D printed models of pediatric aortic arch anomalies (vascular rings and pulmonary artery slings)	Philips IntelliSpace Portal (Philips Healthcare, Best, The Netherlands) Autodesk MeshMixer (Auto- desk, Inc., San Rafael, CA, USA)	Dremel 3D Idea Buidler (Dremel, Mount Prospect, IL, USA).	Polylactic acid (PLA).	3D printed models significantly increased the interventional group’s objective tests assessing anatomy of rings and slings (*p* = 0.001), diagnosis of rings and slings (*p*< 0.001) and treatment of rings and slings (*p*< 0.001). While the total improvement from pre- to post-test scores did not reach statistical significance, the intervention group received the highest average score increase than the control group after the lecture (2.6 vs. 1.8, *p* = 0.084).
Krishnasamy et al. [34]	Cross-sectional study	Thirty doctors, including four cardiothoracic surgeons, 12 cardiologists, five radiologists and nine surgical registrars. All participants were exposed to 3D printed models and asked to complete a questionnaire.	CT	3D printed models of the normal heart	BioModroid	ZCorp Z450 and Stratasys multi-material Connex500	Rubber (cardiac chambers), Tango Plus- synthetic rubber-like material (cardiac muscle), plastic (outer structures).	93% rated the model accuracy as good and above; 64% of the clinicians evaluated this model as an excellent teaching tool for anatomy class. As a visual aid for surgery and planning of treatment, 90% and 74% scored the model to be above average, respectively; 70% of the clinicians scored the model as above average for training purposes. Overall, this 3D printed heart model was rated as excellent (33%), good (50%), and average (17%).
Loke et al. [20]	Randomized controlled trial	Thirty-five pediatric residents with similar previous clinical exposure to Tetralogy of Fallot (ToF). Participants were randomized into control (2D drawings of ToF, *n* = 17) and interventional (3D printed models, *n* = 18)	CT, MRI and 3D echocardiography	3D printed models of a normal infant heart, an adult ToF (repaired) and an infant ToF (unrepaired).	Mimics (Materialize, Leuven, Belgium)	NR	NR	3D printed models gave higher composite learner satisfaction scores (*p* = 0.03). The 3D printed model group also had higher self-efficacy aggregate scores, but the difference was not statistically significant (*p* = 0.39). No significant difference in pre- and post-test scores was found in knowledge acquisition between 2D images and 3D printed models (*p* > 0.05).
Valverde et al. [35]	Non-randomized cross-over study	One hundred and twenty-seven participants (consultants in pediatric cardiology, fellows in pediatric cardiology, and medical students) were randomly distributed into four groups (Groups 1–4) and shown as specific crisscross heart cases (Cases 1–4). Conventional imaging teaching and 3D printed models were presented to each group.	Echocardiography and CMR	Crisscross heart is one of the most complex and extremely rare forms of congenital heart disease. All 3D printed models were based on the four patient-specific CMR datasets. Average printing time: 9.0 ± 1.6 h Average cost of 3D printed model: USD$98 ± 7.5	ITK-SNAP software, version 3.8.0 (University of Pennsylvania, Philadelphia, PA, USA)	3D printer BQ Witbox 1 (Mundo Reader S.L., Madrid, Spain). Models were printed with a 0.2 mm layer height supporting line distance of 5 mm and an overhang angle of 60°.	Filaflex 82A, a flexible polyurethane filament	3D printed models significantly improved crisscross heart anatomy (*p* < 0.001). The increase in the questionnaire marks was statistically significant across all academic groups (*p* < 0.05). Ninety-four percent (120) and 95.2% (121) of the participants agreed or strongly agreed, respectively, that 3D models helped them to better understand the medical images of cardiac anatomy. The average score of overall satisfaction for 3D printing was 9.1 out of 10, with students providing the highest scores than clinical fellows (9.7 vs. 8.6).
White et al. [36]	Randomized controlled trial	Sixty pediatric and emergency medicine residents participated in 2 studies based on pathology (VSD and ToF). Twenty-six were in a VSD study (12 in the control group and 14 in the intervention group). Thirty-four were in the ToF study, 17 each in control and interventional groups. Each group received a 20-min lecture on VSD, with the interventional group receiving 3D printed models.	NR	Four cases, including VSD and ToF	Philips IntelliSpace Portal (Philips Healthcare, Best, The Netherlands)	Dremel 3D Idea Builder (Dremel, Mount Prospect, IL, USA)	Polylactic acid (PLA)	There does not seem to be an added benefit for understanding VSDs (the control group scored higher than the intervention group [*p* = 0.012), but there is for ToF (the interventional group scored higher than the control group, *p* = 0.037). This is likely due to the increased complexity of the lesion and difficulty visualizing spatial relationships in CHD with multiple components.
Tan et al. [37]	Randomized controlled trial	One hundred and thirty-two nursing students in congenital heart surgery were randomly divided into the 3D printing group (*n* = 66) or traditional group (*n* = −66). Both groups were exposed to the same teaching environment with 3D printed model incorporated into the 3D printing group.	One hundred and thirty-two nursing students in congenital heart surgery were randomly divided into the 3D printing group (*n* = 66) or traditional group (*n* = −66). Both groups were exposed to the same teaching environment with 3D printed model incorporated into the 3D printing group.	NR	3D printed model of a patient-specific case of ASD	NR	NR	NR	In the classroom assessment of theoretical knowledge, the 3D printing group scored significantly higher than the traditional group, with corresponding sores being 7.08 ± 1.66 and 6.56 ± 1.17, respectively (*p* = 0.02). The 3D printing group scored significantly higher than the traditional group in the self-evaluation of comprehensive ability and teaching satisfaction score (*p* < 0.05). Similarly, the 3D printing group scored significantly higher than the traditional group in the critical thinking ability assessment (*p* < 0.05).
Biglino et al. [38]	Cross-sectional study	One hundred nurses (65 pediatric cardiac nurses and 35 adult cardiac nurses) attended a training course about CHD.	NR	Nine models were created, including: a healthy heart, repaired transposition of the great vessels, ToF, pulmonary atresia with an intact ventricular septum and palliated hypoplastic left heart syndrome.	NR	NR	NR	The majority of the nurses indicated that 3D printed models helped them to understand cardiac anatomy (86%), spatial orientation (70%) and anatomical complexity after treatment (66%). The 3D printed models were rated as useful by the cardiac nurses, with an average score of 5.1 out of 7.
Liang et al. [39]	Cross-sectional study	Eight CHD cases were 3D printed with clinical values assessed by two groups: one group including 40 experts (20 cardiac surgeons and 20 sonographers); another group consisting of 40 3rd year postgraduate students.	CT	Diagnostic value of 3D printed CHD models, which were printed with blood pool and myocardium models. Eight types of CHD were classified as simple (PDA, CoA and VSD) and complex (ccTGA, DORV, WA, CAF and ToF).	Mimics (Materialize, Leuven, Belgium)	SLA (Shaanxi Hengtong Intelligent Machine Co., Ltd., Xi’an, China). Time and cost for 3D printing and post-processing: 5.88 h and $41 for blood pool models, and 6.25 hr and $42 for myocardial models.	Rigid white resin	3D printing improved the diagnostic rate in both the expert and student groups, with the average value being 95.9% and 91.6% after using 3D printed models vs. 88.75% and 60% before using 3D printed models (*p* = 0.000–0.001). 3D printed models were found to be more important in diagnosing complex CHD than simple CHD, and the demand was stronger in students than from experts. Myocardial models were found to be more advantageous in DORV and VSD by demonstrating the location and structure of the lesions, while blood pool models were more effective in assessing other CHD diseases.

ASD—atrial septal defect, CAF—coronary artery fistula, CHD—congenital heart disease, CoA—coarctation of aorta, CT—computed tomography, ccTGA—corrected transposition of the great arteries, d-TGA—d-transposition of the great arteries, DORV—double outlet right ventricle, HLHS—hypoplastic left heart syndrome, MRI—magnetic resonance imaging, NR—not reported, PDA-patent ductus arteriosus, ToF—tetralogy of Fallot, VR—virtual reality, VSD—ventricular septal defect, WS—William syndrome.

**Table 2:** The comparison of the results of critical thinking ability assessment between the two groups of nursing students [point, ([x^—^ ± s]). Reprinted with permission under the open access from Tan et al. [37].

Observation Indices	3D Printing Group *n* = 64	Traditional Group *n* = 66	T Value	*p* Value
Total score	(299.98 ± 1.20)	(282.61 ± 0.67)	12.586	<0.01
Truth-seeking	(42.00 ± 0.58)	(40.67 ± 0.29)	2.046	0.02
Open-mindedness	(43.73 ± 0.52)	(40.83 ± 0.26)	4.913	<0.01
Analytical ability	(42.59 ± 0.42)	(39.95 ± 0.23)	5.449	<0.01
Systemic ability	(44.56 ± 0.27)	(40.30 ± 0.23)	11.926	<0.01
Confidence in thinking	(44.38 ± 0.24)	(41.16 ± 0.28)	8.878	<0.01
Curiosity	(42.21 ± 0.36)	(40.73 ± 0.42)	2.690	<0.01
Cognitive maturity	(40.52 ± 0.45)	(38.97 ± 0.35)	2.716	<0.01

**Table 3:** Clinical value of 3D printed models in surgical planning of cardiac procedures.

Author	Study Design	Sample Size and Participants	Original Data Source	Application in CVD	Image Processing Software	3D Printer/ Printing Parameters	3D Printing Material	Key Findings
Valverde et al. [47]	Prospective multi-center study	Forty patients with complex CHD. Patients took part in the surgical planning meetings alongside surgeons, cardiologists and radiologists.	CT and MRI	3D printed models of complex CHD: DORV: 19 and other types of CHD: 21	ITK snap software & Meshmixer version 11.0.544	BQ Witbox, Spain. A 0.8 mm outer shell was added outside of the blood pool interface.	Fused deposition modeling with the use of polyurethane filament	The surgical planning of 21 cases (52% of total cases, confidence interval 38.5–70.7%) was not influenced by 3D printed models, and the surgical decision remained based on the patient’s imaging. The surgical decision in the other 19 cases was changed by 3D printed models (47.5% of total cases, confidence interval of 29.6–61.5%). A 53.6% change in the surgical plan after using 3D printed models.
Chen et al. [48]	Cross-sectional study	Five patients with pulmonary atresia (PA) with VSD or major aortopulmonary collateral arteries (MAPCA) had a 3D model of their heart printed prior to the unifocalization procedure.	Echocardiography and CT	Five patients with PA/VSD/MAPCA were subjected to 3D printing of the heart before the single-stage unifocalization procedure. The 3D models were also visualized in the operating room using VR glasses.	3Matic (Materialise, Leuven, Belgium) & ITKsnap 3X. Microsoft HoloLens for mixed reality	NR	NR	The 3D models aided surgeons in visualizing spatial relationships between the major vessels and the precise location of each MAPCA up to a few mm in accuracy. This was beneficial to the surgical strategy and pre-operative planning as surgeons had a better comprehension of the procedure prior to the surgery. VR simulation enabled surgeons to have a comprehensive understanding of both spatial anatomy and surgical procedure.
Gomez-Ciriza et al. [22]	Cross-sectional study with seven years of experience	One hundred thirty-eight heart models were generated. In addition, 13 cardiac surgeons and 30 pediatric cardiologists were introduced to 3D-printed models of congenital heart diseases.	CT and MRI	Patients with CHD were recruited from several hospitals. One hundred and thirty-eight low-cost 3D printed models, with 83 used for surgical planning, 48 for interventional planning and 7 for the interventional simulator	ITK-SNAP and Cura. Average time for printing and cleaning: 13.5 h Average cost of each model: EUR85.7	BQ Witbox Fused Deposition Modeling 3D printer. Z-axis resolution: 0.02 mm. Heart models were printed with 0.15 mm Z resolution.	Filaflex	With the use of 3D printed models, the surgical planning was modified in 47.5% of the patients compared to the original surgical plan, which used only clinical information and medical imaging. 3D printed models were scored useful for communicating with parents and patients. Further, they were allowed to plan specific interventions and develop new strategies for treating CHD.
Guo et al. [49]	Cross-sectional study	Seven HOCM patients who received surgical management had 3D models of their hearts printed.	CT	Surgical management of hypertrophic obstructive cardiomyopathy (HOCM)	Mimics 19.0 (Materialise, Leuven, Belgium)	Objet350 Connex3 (Stratasys Ltd., Eden Prairie, MN, USA)	VeroMagenata Veroclear VeroCyan Tango material	The pre- and postoperative echocardiographic evaluation revealed that left ventricular outflow tract (LVOT) obstruction was adequately relieved (82.71 ± 31.63 to 14.91 ± 6.89 mmHg, *p* < 0.001) and septal thickness was reduced from 21.57 ± 4.65 to 17.42 ± 5.88 mm (*p* < 0.001). Patients were highly satisfied with the use of 3D printed models in explaining the disease.
Kiraly et al. [24]	A cross-sectional study amongst the multidisciplinary and intraoperative team	Fifteen patients with complex congenital cardiac conditions for cardiac surgery.	CT	3D prototypes of the heart-great vessels for 15 case scenarios undergoing surgical management.	Mimics (Materialise, Leuven Belgium) and 3D Slicer	Stratasys J750 resin-based 3D printer (Stratasys, Eden Prairie, MN, USA). Hollow models with 2–3 mm wall thickness.	VeroMagenta, TangoPlus and HeartPrint Flex	3D printed models refined diagnosis in 9 out of 15 cases, while in 13 cases (86.66%), redo-operations were planned after reviewing 3D printed models. 3D printed models revealed new anatomic findings in 60% of cases, of which 3D printed models offered an alternative surgical plan in 3 cases. 3D printed models enabled surgical simulation and pre-procedural planning for the shape and size patches.
Ryan et al. [50]	Cross-sectional study with a single center of 3 years of experience	One hundred sixty-four models were printed for various purposes with operating room length and mortality/morbidity compared between the standard of care (SoC) and the use of 3D printed models.	CT & MRI	79 3D printed models of CHD (different types) were used for surgical planning and comparison between the methods.	Mimics (Materialise, Leuven, Belgium)	zPrinter 650 (3D Systems, Rock Hill, SC, USA).	Gypsum-based resin	3D printed models reduced operating length of time (mean 202.7 vs. 229.3 min, *p* = 0.674), reduced 30-day mortality, and readmission rate for patients (60% vs. 71.6%) when compared to SoC, although these comparisons did not reach statistical significance.
Zhao et al. [51]	Cross-sectional study	Twenty-five patients with complex DORV were divided into two groups: the control group (17) and the 3D printing group (8).	CT	Clinical value of 3D printed models in planning preoperative strategies for surgical repair of patients with complex DORV.	Mimics (Materialise, Leuven, Belgium)	ZPrinter 650	Polylactic acid	The 3D printing group had significantly reduced the mechanical ventilation time (56.43 vs. 96.76 h, *p* = 0.040) and intensive care unit time (99.04 vs. 166.94 h, *p* = 0.008) than the control group. The operative, cardiopulmonary bypass and aortic-clamping times were shorter in the 3D printing group than in the control group, with no significant difference (*p* > 0.05 for all).
Ghosh et al. [52]	Retrospective study over a period of 3 years	One hundred and twelve patient-specific 3D printed models of CHD, with 16 used for teaching purposes and 96 for pre-operative planning.	MRI and CT	How 3D printing service is incorporated into routine pre-procedural care of pediatric patients with acquired or congenital CHD.	Mimics 20.0 (Materialise, Leuven, Belgium)	Objet500 Connex 3 & J750, Stratasys(Eden Prairie, MN, USA)	Rigid photopolymers with a minimum thickness of 1.0 mm and flexible rubber photopolymers with a minimum thickness of 0.75 mm.	The most common applications of 3D printed models were to evaluate intracardiac anatomy for complex biventricular repair (31%) and preoperative evaluation of extracardiac pathology (20%), followed by repair of multiple ventricular septal defects (12%). In about one-third (34%) of cases, 3D printed models were requested prior to patients undergoing CT or MRI imaging. 3D printed models can be incorporated into the pre-procedural care of patients with CHD.
Russo et al. [53]	Cross-sectional multi-center study	Thirteen patients with aortic stenosis at a high risk of coronary artery obstruction (CAO).	CT	3D printed models to simulate transcatheter aortic valve replacement (TAVR) procedures for improving CAO risk assessment and prediction of complications and procedural safety.	Mimics 20.0 (Materialise, Leuven, Belgium)	NR	Heart-Print Flex technology- a combination of flexible and rigid polymers.	TAVR was successfully stimulated in all 13 printed models with a high risk of CAO. The 3D model simulations correlated well with clinical outcomes by accurately replicating specific anatomy and procedural outcomes. 3D printed models serve as a useful simulation platform in high-risk TAVR patients to predict complications.

Abbreviations are the same as shown in Table 1.

**Table 4:** Clinical application of 3D printed models in simulation of surgical or interventional procedures.

Author	Study Design	Sample Size and Participants	Original Data Source	Application in CVD	Image Processing Software	3D Printer/ Printing Parameters	3D Printing Material	Key Findings
Fan et al. [54]	A mix of retrospective and prospective study	Retrospective group of 72 patients with LAA occlusion guided by 3D TEE imaging. Prospective group of 32 patients with LAA occlusion guided by 3D printed models.	3D transesophageal echocardiography (TEE)	3D printed models in LAA device selection and assessment of procedural safety and efficacy.	Mimics 19.0 (Materialise, Leuven, Belgium)	Objet350 Connex 3 (Stratasys). Hollow wall thickness: 1 mm. Printing resolution: 32 μm.	Agilus A30 Clear (Stratasys)	The implantation success was 100% and 93.1% in retrospective and prospective groups. The prospective group with the use of 3D printed models achieved shorter procedural time, few devices used for the procedure (*p* < 0.05 for all) with no procedure complications. There were no major adverse cardiac events or mortality in the prospective group at a mean follow-up of 9.4 months, while there were three major adverse events and nine deaths in the retrospective group at a mean follow-up of 3 years.
Hell et al. [55]	Cross-sectional study	Twenty-two patients underwent LAA occlusion	TEE and CT	Prediction of device size and compression for LAA occlusion	3D Slicer 5–6 h, 20 EUR	Ultimaker 2 (Ultimaker, B.V., Geldermalsen, The Netherlands)	Silicon rubber	There was 95% agreement between 3D printed model-based sizing and the finally implanted device sizes, while the agreement between CT- and TEE-based device sizes was only 77% and 45%, respectively. The 3D printed models correlated well with the prediction of device compression (*p* = 0.003).
Li et al. [56]	Randomized controlled trial	Twenty-one patients in the 3D printing group with 3D printed model guiding occlusion device selection. Twenty-one patients in the control group with an occlusion device selected by TEE, CT and angiography.	TEE and CT	LAA occlusion device selection.	Mimics 17.0 (Materialise, Leuven, Belgium)	NR	NR	The occlusion procedure was successful in both groups. The procedure time, contrast volume, and costs were 96.4 ± 12.5 vs. 101.2 ± 13.6 min, 22.6 ± 3.0 vs. 26.9 ± 6.2 mL, and 12,671.1 vs. 12,088.6 USD for 3D printing and control groups, respectively. The radiation dose was significantly lower in the 3D printing group than that in the control group (561.4 ± 25.3 vs. 651.6 ± 32.1 mGy, *p* < 0.05).
Conti et al. [57]	Case-control study	Twenty patients: 6 with LAA leak and 14 control patients without LAA leak.	CT	3D printed models in LAA size to prevent LAA leak.	ITK-SNAP0	Form 2 Desktop printer (Formlabs, Inc., Somerville, MA, USA)	NR	Compared to 3D printed models, the device sizes based on traditional imaging analysis were unestimated in 11 patients (55%), agreed with the implanted sizes in 7 (35%) and overestimated in 2 (10%) cases. Of 8 cases with an implant device of 22 mm, 75% of the 3D printed models matched the implanted size.
Goitein et al. [58]	Cross-sectional study	Twenty-nine patients underwent LAA occlusion.	TEE and CT	3D printed models to size LAA occluder. Two types of occluder were used: AMPLATZER: *n* = 12 WATACHMAN: *n* = 17	Comprehensive Cardiac Analysis (v 4.5, Philips Healthcare)	Objet (Rehovot, Israel)	TangioPlus FLX930 (Stratasys, Germany)	A high correlation was found between 3D printed models and the inserted device size for the AMPLATZER occluder (*p* = 0.001) but was a poor correlation between 3D printed models and the WATCHMAN device (*p* = 0.203).
Torres and Luccia [59]	Cross-sectional study (prospective controlled single-center trial)	Control group: 5 residents operated on 30 patients; Training group: 5 residents operated on 25 patients.	CT	Twenty-five aneurysms were 3D printed for training and simulation of EVAR procedure when compared to the traditional training approach.	TeraRecon iNtuition Unlimited (Aquarius v 4.3, TeraRecon, CA, USA)	Connex350 (Stratasys), Formlabs Form 1 and MakerFiveot	Five materials used for 3D printing: Rubber FLX930 Plastic RGD810 TangoPlus +Vero Clear Shore 60 Resin and PLA in silicone	Use of the3D models printed with flexible resin and silicone produced the best results. Patient-specific training based on 3D printed models reduced fluoroscopy time by 30% (33 vs. 48 min), total procedure time by 29% (207 vs. 292 min), the volume of the contrast medium by 25% (65 vs. 87 mL) and time for cannulation by 52% (3 vs. 6 min) when compared to the control group (*p* < 0.05 for all), respectively.
Karkkainen et al. [60]	Cross-section study (prospective pilot study)	22 participants: 20 trainees: Group A: 13 experience in <20 EVAR Group B: 7 experience in >20 EVAR procedures.	CT	Use of a 3D printed AAA model in 20 trainees and two experienced operators to perform EVAR simulations	Mimics (Materialise, Leuven, Belgium	Objet500 Connex3 (Stratasys). Models were printed with three layers: 3 mm, 3 mm and 1 mm for rigid inner and flexible outer layers and luminal side, respectively.	VeroClear and Agilus	The mean procedure time was 37 ± 12 min for all 22 simulations. Experienced trainees completed the simulation procedures with significantly lower time and fluoroscopy time than inexperienced trainees (*p* < 0.05).
Kaufamann et al. [61]	Cross-sectional study	27 interventional radiology (IR) procedures with 54 3D printed models.	CT	Fifty-four vascular models were printed with clear and transparent flexible resin for comparison of IR procedures.	Image J	Form 3 (Formlabs). Printing resolution: 0.1 mm.	Standard clear (transparent but rigid) and flexible resin (transparent and flexible).	Of the 216 measurements in the aorta and aortic branch diameters, there were no significant differences in all measurements between the original CT and clear and flexible resin models (*p* > 0.05). Printing accuracy was excellent for both materials (<0.5 mm). Printing success was 85.2% and 81.5% for standard clear and flexible resin, respectively.
Sheu et al. [62]	Randomized controlled trial	Forty-nine medical students were enrolled, with 26 assigned to 3D printed vascular model and 23 to the control group (commercial simulator).	CT	3D printed model training medical students in performing femoral artery (FA) access.	TeraRecon Aquarium Intuition (TeraRecon Inc., Foster City, CA, USA)	Formlabs Form 2 SLA. The model was hollowed to 0.75 mm wall thickness.	Resin	Prior to simulation, 76.9% of trainees in 3D printing and 82.6% of trainees in the control group did not feel confident performing FA access. After the simulation, both groups agreed that the model increased their confidence in performing FA by 2 Likert points (*p* < 0.01). The confidence increase in the 3D printing group was non-inferior to that in the control group (*p* < 0.001).
Goodie et al. [63]	Cross-sectional study	Thirty medical students were invited to evaluate the efficacy of 3D printed vascular models.	CT and MRI	Five aorta and vascular models were created to simulate interventional radiology (IR) procedures.	Osirix Lite Library	Ultimaker 2 and Lulzbot Taz ^TM^	PLA and NinjaTek Cheetah ^TM^	3D printed models served as a supplementary tool to traditional teaching for simulation and rehearsal of IR procedures.
Yoo et al. [64]	Cross-sectional study	Eighty-one surgeons or surgical trainees were presented with the 3D printed models and subsequently performed simulated surgical procedures under guided supervision.	CT and MRI	Hands-on surgical training using 3D printed models of CHD (DORV and HLHS) in simulation of congenital heart surgeries.	Mimics (Materialise, Leuven, Belgium) Average cost per model: $60	Objet Connex 260 printer. A shell thickness of 1.2–1.8 mm was added to the outer surface of the segmented model.	TangoPlus FullCure resin and VeroWhite	Fifty attendees participated in the survey after training sessions. 3D printed models were considered as acceptable quality (88%) or manageable (12%) aid in surgical practice. Further improvements were suggested, including using material more akin to human cardiac valves.
Brunner et al. [65]	Cross-sectional study	Nineteen medical students and doctors participated in the hands-on training program.	CT	Hands-on training on simulation of interventional cardiology procedures on common CHD models.	Mimics (Materialise, Leuven, Belgium)	Agilista 3200W Polyjet 3D printer	Silicone rubber	Practicing on 3D printed models led to a significant reduction in the mean fluoroscopy time. All participants gave 3D printed models very positive ratings as a training tool for the simulation of interventional cardiac procedures.
Rynino et al. [66]	Cross-sectional study	Eleven models of aortic dissection cases were printed using different materials and distributed into four groups: autoclave 121 °C sterilization, plasma sterilization, gas sterilization, 105 °C autoclave sterilization.	CT	Effect of sterilization methods on the geometric changes of the 3D printed aortic template.	3D Slicer	Raise3D Pro 2 printer (Raise3D, Irvine, CA, USA) & Form 2 (Formlabs, Somerville, MA, USA). 1.5 mm was added to the segmented aortic wall.	Polylactic acid (PLA), nylon, polypropylene (PP), polyethylene terephthalate glycol (PETG), and a rigid and flexible photopolymer resin.	3D printed models made from PLA, PETG and PP were deformed during sterilization at high temperatures (autoclave 121 °C). However, 3D-printed models made with nylon or flexible and rigid resin did not undergo filament deformities during high-temperature sterilization. All mean geometry differences were less than 0.5 mm.

Abbreviations are the same as shown in Table 1.

## Data Availability

Not applicable.
